# Economic Cost of Injury — United States, 2019

**DOI:** 10.15585/mmwr.mm7048a1

**Published:** 2021-12-03

**Authors:** Cora Peterson, Gabrielle F. Miller, Sarah Beth L. Barnett, Curtis Florence

**Affiliations:** 1 Division of Injury Prevention, National Center for Injury Prevention and Control, CDC.

Unintentional and violence-related injuries, including suicide, homicide, overdoses, motor vehicle crashes, and falls, were among the top 10 causes of death for all age groups in the United States and caused nearly 27 million nonfatal emergency department (ED) visits in 2019.*^,^^†^ CDC estimated the economic cost of injuries that occurred in 2019 by assigning costs for medical care, work loss, value of statistical life, and quality of life losses to injury records from the CDC’s Web-based Injury Statistics Query and Reporting System (WISQARS).^§^ In 2019, the economic cost of injury was $4.2 trillion, including $327 billion in medical care, $69 billion in work loss, and $3.8 trillion in value of statistical life and quality of life losses. More than one half of this cost ($2.4 trillion) was among working-aged adults (aged 25–64 years). Individual persons, families, organizations, communities, and policymakers can use targeted proven strategies to prevent injuries and violence. Resources for best practices for preventing injuries and violence are available online from CDC’s National Center for Injury Prevention and Control.^¶^

The economic cost estimate for injuries that occurred in 2019 uses the societal perspective, including tangible and intangible costs to multiple payers, and a 1-year time horizon (period over which costs are assessed) for nonfatal injuries. Costs are presented in 2019 U.S. dollars (USD). WISQARS nonfatal injury counts are hospital ED injury visits from the nationally representative National Electronic Injury Surveillance System – All Injury Program. WISQARS fatal injury counts are from CDC’s National Vital Statistics System mortality data.

Medical and work loss costs (*1*,*2*) were adjusted for patient clinical and demographic characteristics, including comorbidities, sex, and age, and modified to 2019 USD.** Medical costs were assigned to WISQARS records by injury outcome (fatal or nonfatal), mechanism (e.g., fall), intent (e.g., unintentional), and place of death (e.g., inpatient hospital) or ED visit disposition (treated and released or hospitalized, including transferred). Work loss costs for nonfatal injuries were assigned by injury mechanism and ED visit disposition to injured persons of all ages; this approach assumes injured children and older adults incur lost productivity among working-aged adult caregivers. Aggregated medical and work loss costs (e.g., combined intents by mechanism or combined mechanisms by ED visit disposition) from reference sources were assigned when specific estimates by intent or mechanism were not available.

The cost of injury mortality includes value of statistical life, a monetary estimate of the collective value placed on mortality risk reduction as derived in research studies through revealed preferences (e.g., observed wage differences for dangerous occupations) or stated preferences from surveys of individual persons’ willingness to pay for mortality risk reduction (*3*). Value of statistical life estimates were assigned by decedent age: 0–17 years, $16.9 million (*4*); 18–65 years, $10.7 million (*3*); and values descending from $6 million (aged 66 years) to $410,000 (aged ≥100 years), reflecting the estimate for persons aged 18–65 years adjusted for older adults’ decreasing general life expectancy and baseline quality of life. Cost of nonfatal injury morbidity includes quality of life losses measured in terms of quality-adjusted life years (QALY; 1 QALY equals 1 year of perfect health) (*5*) and valued at $540,000 per QALY (*3*). Injury count, rate per 100,000 population, cost by type (medical, work loss, value of statistical life, and quality of life loss), and total cost are reported by intent, sex, and age group. All reported data can be queried online using WISQARS. This activity was reviewed by CDC and was conducted consistent with applicable federal law and CDC policy.^††^

In 2019, the economic cost of injury was $4.2 trillion, including $327 billion in medical care, $69 billion in work loss, and $3.8 trillion in value of statistical life and quality of life losses (Table). The economic costs were $2.2 trillion for fatal injuries and $2.0 trillion for nonfatal injuries. The number of injury deaths and associated economic cost were higher among males (169,628 and $1.6 trillion, respectively) than among females (76,413 and $607 billion, respectively). The cost of nonfatal injury was similar for males and females ($1 trillion). Except for nonfatal self-harm, the age-adjusted rate, number, and economic cost for all injury outcomes (fatal and nonfatal) and intents (unintentional, homicide or assault) were higher for males than for females.

**TABLE:** Number, rates, and estimated costs* of injuries, by outcome, intent, sex, and age group — United States, 2019

Outcome and intent	Total	Sex		Age group, yrs				
		Male	Female	0–14	15–24	25–44	45–64	≥65
Total cost	4,208,579	2,609,647	1,598,906	396,491	512,206	1,213,049	1,180,231	905,945
Medical	326,774	179,673	147,094	33,151	38,522	82,724	77,607	94,514
Work loss	68,729	37,085	31,642	7,472	8,751	18,165	16,758	17,545
Value of statistical life and quality of life	3,813,077	2,392,888	1,420,170	355,868	464,932	1,112,160	1,085,866	793,886
**Fatal injuries**								
**All intents^†^**								
No. of deaths	246,041	169,628	76,413	5,590	23,051	75,488	70,453	71,435
Rate^§^	71.1	102.8	40.8	9.2	54.0	86.2	84.6	132.1
Costs	2,186,049	1,578,711	607,338	94,559	267,218	808,334	754,570	261,368
Medical	3,786	2,226	1,560	88	205	612	723	2,158
Value of statistical life	2,182,263	1,576,484	605,778	94,471	267,013	807,722	753,847	259,210
**Unintentional**								
No. of deaths	173,040	112,720	60,320	3,907	11,755	48,586	48,251	60,527
Rate^§^	49.2	68.2	31.3	6.5	27.5	55.5	57.9	112.0
Costs	1,447,643	1,006,091	441,552	66,086	134,498	520,291	516,874	209,894
Medical	3,265	1,834	1,430	58	114	421	588	2,084
Value of statistical life	1,444,378	1,004,257	440,122	66,028	134,384	519,870	516,286	207,810
**Homicide**								
No. of deaths	19,141	15,264	3,877	893	4,774	8,787	3,614	1,071
Rate^§^	6.0	9.6	2.4	1.5	11.2	10.0	4.3	2.0
Costs	209,019	167,502	41,517	15,109	55,581	94,105	38,710	5,514
Medical	204	170	34	18	47	84	40	14
Value of statistical life	208,816	167,332	41,484	15,092	55,533	94,021	38,670	5,500
**Suicide**								
No. of deaths	47,511	37,256	10,255	546	5,954	15,584	16,250	9,173
Rate^§^	13.9	22.4	6.0	0.9	14.0	17.8	19.5	17.0
Costs	463,193	359,092	104,102	9,235	70,567	166,836	173,946	42,610
Medical	252	179	73	7	39	87	71	47
Value of statistical life	462,941	358,912	104,029	9,227	70,528	166,749	173,875	42,562
**Nonfatal injuries^¶^**								
All intents**								
No. of injuries	25,933,780	13,973,305	11,960,119	4,102,128	3,842,368	7,275,609	5,929,789	4,778,380
Rate^§^	7,881.5	8,699.3	7,037.9	6,772.5	9,001.2	8,305.5	7,116.6	8,839.3
Costs	2,022,531	1,030,936	991,568	301,932	244,988	404,716	425,661	644,577
Medical	322,988	177,447	145,534	33,063	38,317	82,112	76,884	92,356
Work loss	68,729	37,085	31,642	7,472	8,751	18,165	16,758	17,545
Quality of life	1,630,814	816,404	814,392	261,397	197,919	304,438	332,019	534,676
**Unintentional**								
No. of injuries	23,973,103	12,865,348	11,107,407	3,953,061	3,319,180	6,412,723	5,556,825	4,727,632
Rate^§^	7,256.4	8,001.0	6,484.9	6,526.3	7,775.5	7,320.5	6,669.0	8,745.4
Costs	1,840,193	920,286	919,881	291,077	199,765	324,816	386,194	637,937
Medical	285,673	154,120	131,548	30,854	28,092	65,722	69,641	91,250
Work loss	62,889	33,896	28,991	7,081	7,124	15,763	15,554	17,341
Quality of life	1,491,631	732,271	759,342	253,143	164,549	243,330	300,999	529,345
**Assault**								
No. of injuries	1,421,988	854,340	567,648	101,918	348,467	659,136	277,316	33,403
Rate^§^	452.2	537.8	366.7	168.3	816.3	752.4	332.8	61.8
Costs	149,534	92,853	56,680	8,533	35,651	66,450	33,297	5,352
Medical	23,689	17,116	6,573	1,046	5,883	11,386	4,625	609
Work loss	2,605	1,821	784	125	605	1,229	555	80
Quality of life	123,240	73,916	49,324	7,362	29,163	53,836	28,117	4,663
**Self-harm**								
No. of injuries	460,416	186,954	273,455	46,429	157,635	158,489	82,642	15,221
Rate^§^	147.9	118.1	178.9	76.7	369.3	180.9	99.2	28.2
Costs	26,705	12,528	14,176	2,277	8,169	10,020	5,089	1,150
Medical	12,601	5,340	7,260	1,157	4,127	4,425	2,432	459
Work loss	3,104	1,259	1,845	266	994	1,098	627	120
Quality of life	11,000	5,929	5,071	854	3,047	4,497	2,031	571

**Abbreviation:** USD = U.S. dollars.

* In millions of 2019 USD.

^†^ Fatal all intents estimates include injuries with legal intervention intent, undetermined intent, unknown sex, and unknown age.

^§^ Per 100,000. Age-adjusted rate is presented for “Total,” “Male,” and “Female” columns.

^¶^ Nonfatal injuries are an estimated number of hospital visits for injury care that start in an emergency department (with disposition treated and released, transferred, or hospitalized; visits with observed, left against medical advice, and unknown disposition were not included) based on a nationally representative probability sample of hospitals.

** Nonfatal all intents estimates include injuries with legal intervention intent, unknown sex, and unknown age. Nonfatal assault, self-harm, and legal intervention include cases that are confirmed or suspected; all other cases are considered unintentional.

Economic cost was highest for persons aged 25–44 and 45–64 years ($1.2 trillion each), followed by those aged ≥65 years ($906 billion), 15–24 years ($512 billion), and 0–14 years ($396 billion). Although the injury fatality rate was highest among those aged ≥65 years (132.1 per 100,000; mostly unintentional [112.0]), the economic cost of fatal injuries was higher for those aged 25–44 years ($808 billion) and 45–64 years ($755 billion) than for those aged ≥65 years ($261 billion) because of higher value of statistical life cost. The economic cost of suicide deaths was highest among those aged 25–44 years ($167 billion) and 45–64 years ($174 billion). The economic cost of deaths from homicide was highest among those aged 25–44 years ($94 billion), followed by those aged 15–24 years ($56 billion). The economic cost of nonfatal injuries was highest among those aged ≥65 years ($645 billion), primarily because of quality of life loss costs from unintentional injuries, followed by those aged 45–64 years ($426 billion), 25–44 years ($405 billion), 0–14 years ($302 billion), and 15–24 years ($245 billion). The economic cost of nonfatal injuries from assault and self-harm were highest among those aged 25–44 years ($66 billion and $10 billion, respectively).

## Discussion

This report used injury incidence data to estimate the economic cost of injuries that occurred in the United States during 2019. Economic cost was highest among working-aged adults, highlighting that injuries during the most productive part of people’s lives result in a high societal cost. These findings highlight the need for targeted prevention strategies to achieve long-term value, or even cost-savings, by preventing injury morbidity and mortality through addressing the causes of unintentional and violence-related injuries at the individual, family, organizational, and community levels.

The 2019 economic cost of injuries ($4.2 trillion) is more than six times as high as a comparable estimate in 2013 ($671 billion) (*6*,*7*). Even though the number of nonfatal ED injury visits in 2019 was approximately 15% lower than it was in 2013, the 2019 nonfatal injury economic cost ($2.0 trillion) is more than four times as high as the 2013 estimate ($457 billion) (*6*), primarily because of including the cost of diminished quality of life. The 2019 fatal injury economic cost ($2.2 trillion) is substantially higher than the similar estimate in 2013 ($214 billion) (*7*). This difference reflects a 28% higher number of injury deaths in 2019 and mortality cost based on value of statistical life, which represents a value that is approximately 10 times as high as the value attributed to mortality based on foregone employment compensation, which was used in the previous estimate. 

The findings in this report are subject to at least five limitations. First, the economic cost of nonfatal injuries is underestimated because only injuries treated in an ED are included (injuries initially treated in urgent care or doctor’s offices not included), other costs such as property damage and criminal justice are not included, and nonfatal costs address only the first year following an injury. The cost of nonfatal injury includes observed medical care and work loss attributable to injuries based on comparing injured patients and non-injured persons during the year following the injured patient’s initial ED visit (*1*,*2*). A 1-year time horizon is appropriate for many injury types but does not address the long-term physical and mental health consequences of some injuries (e.g., traumatic brain injury and violence-related injuries). Second, although injury-related medical care and work loss have costs to specific, identifiable payers (including individual persons, health insurance payors, and employers), the highest cost elements presented here are value of statistical life and quality of life losses; these costs are not readily identifiable through financial transactions and thus not as visible to some stakeholders as are direct costs, such as medical care. Third, although this study aimed for a reasonable use of available value of statistical life data, the relationship between value of statistical life and age (particularly, value of statistical life for older adults) is likely more complex than applied here and would benefit from further direct study (*8*). Fourth, quality of life loss estimates might indirectly capture some work loss; therefore, the nonfatal economic cost estimate might partially double count such costs. Finally, this report provides an initial assessment of the economic cost of injury by intent based on injured person sex and age group. Estimation of injury costs by other demographic and geographic factors within the United States can provide additional meaningful information for injury prevention.

Individual persons, families, organizations, communities, and policymakers can use targeted proven strategies to prevent injuries and violence. Data and resources that can assist in measuring and preventing injuries and violence, including suicide, overdoses, falls, firearm violence, motor vehicle crashes, traumatic brain injury, adverse childhood experiences, youth violence, sexual violence, and intimate partner violence, are available online from CDC’s National Center for Injury Prevention and Control. Opportunities to investigate injury data and costs are available online from WISQARS.

**Summary**

**What is already known about this topic?**

Unintentional and violence-related injuries, including suicide, were among the top 10 causes of U.S. deaths for all age groups and caused nearly 27 million nonfatal emergency department visits in 2019.

**What is added by this report?**

Fatal and nonfatal injury data from CDC’s Web-based Injury Statistics Query and Reporting System were matched to medical care, work loss, value of statistical life, and quality of life loss costs. The estimated U.S. economic cost of injuries in 2019 was $4.2 trillion. More than one half of this cost ($2.4 trillion) was among working-aged adults (aged 25–64 years).

**What are the implications for public health practice?**

Unintentional and violence-related injuries are costly and preventable. Resources for best practices for preventing injuries and violence are available online from CDC’s National Center for Injury Prevention and Control.

## Footnotes

* Data on leading causes of death and years of potential life lost are available from https://wisqars.cdc.gov/data/lcd (Accessed November 29, 2021).

† Data on estimated number of nonfatal emergency department visits for injuries are available from https://wisqars.cdc.gov/data/non-fatal/explore (Accessed November 29, 2021).

†† 45 C.F.R. part 46, 21 C.F.R. part 56; 42 U.S.C. Sect. 241(d); 5 U.S.C. Sect. 552a; 44 U.S.C. Sect. 3501 et seq.

## References

[1] Peterson C, Xu L, Barnett SBL. Average lost work productivity due to non-fatal injuries by type in the USA. Inj Prev 2021;27:111–7. 10.1136/injuryprev-2019-043607 32366517 PMC7609459

[2] Peterson C, Xu L, Florence C. Average medical cost of fatal and non-fatal injuries by type in the USA. Inj Prev 2021;27:24–33. 10.1136/injuryprev-2019-043544 31888976 PMC7326639

[3] Office of the Assistant Secretary for Planning and Evaluation. Guidelines for regulatory impact analysis. Washington, DC: US Department of Health and Human Services, Office of the Assistant Secretary for Planning and Evaluation; 2016. https://aspe.hhs.gov/sites/default/files/migrated_legacy_files//171981/HHS_RIAGuidance.pdf

[4] Office of Child Care, Administration for Children and Families, US Department of Health and Human Services. Child Care and Development Fund (CCDF) program. Final rule. Fed Regist 2016 Sep30;81(190):67438–595. 27726322

[5] Lawrence B, Miller T. Quality of life loss estimation methods for the WISQARS Cost of Injury Module. Calverton, MD: Pacific Institute for Research and Evaluation; 2020. https://www.cdc.gov/injury/wisqars/pdf/PIRE_WISQARS_cost_injury_quality_life_loss_estimation_methods-508.pdf

[6] Florence C, Haegerich T, Simon T, Zhou C, Luo F. Estimated lifetime medical and work-loss costs of emergency department-treated nonfatal injuries—United States, 2013. MMWR Morb Mortal Wkly Rep 2015;64:1078–82. 10.15585/mmwr.mm6438a5 26421663

[7] Florence C, Simon T, Haegerich T, Luo F, Zhou C. Estimated lifetime medical and work-loss costs of fatal injuries—United States, 2013. MMWR Morb Mortal Wkly Rep 2015;64:1074–7. 10.15585/mmwr.mm6438a4 26421530

[8] Aldy JE, Viscusi WK. Adjusting the value of a statistical life for age and cohort effects. Rev Econ Stat 2008;90:573–81. 10.1162/rest.90.3.573

